# Dysfunctional adiposity index as a marker of adipose tissue morpho-functional abnormalities and metabolic disorders in apparently healthy subjects

**DOI:** 10.1080/21623945.2021.1893452

**Published:** 2021-03-16

**Authors:** Juan Reyes-Barrera, Victor H. Sainz-Escárrega, Aida X. Medina-Urritia, Esteban Jorge-Galarza, Horacio Osorio-Alonso, Margarita Torres-Tamayo, Gabriela Leal-Escobar, Carlos Posadas-Romero, Ivan Torre-Villalvazo, Juan G. Juárez-Rojas

**Affiliations:** a Department of Endocrinology, Instituto Nacional de Cardiología Ignacio Chávez, Mexico City, México; b Department of Cardiothoracic Surgery, Instituto Nacional de Cardiología Ignacio Chávez, Mexico City, México; c Department of Cardio-Renal Physiopathology, Instituto Nacional de Cardiología Ignacio Chávez, Mexico City, México; d Department of Nephrology, Instituto Nacional de Cardiología Ignacio Chávez, Mexico City, México; e Department of Nutrition Physiology, Instituto Nacional De Ciencias Médicas Y Nutrición Salvador Zubirán, Mexico City, México

**Keywords:** Adipose tissue, Dysfunctional adiposity index, cardiometabolic abnormalities

## Abstract

Compared to body mass index, waist circumference (WC), and adiposity measurements, adipose tissue (AT) morpho-functionality evaluations are better predictors of cardiometabolic abnormalities (CA). The present study establishes a dysfunctional adiposity index (DAI) as an early marker of CA based on adipocytes morpho-functional abnormalities. DAI was established in 340 subjects without cardiovascular risk factors selected from a cross-sectional study (n=1600). Then, DAI was calculated in 36 healthy subjects who underwent subcutaneous AT biopsy. The correlation of DAI with adipocyte morphology (size/number) and functionality (adiponectin/leptin ratio) was analyzed. The DAI cut-off point was identified and its independent association with CA was determined in 1418 subjects from the cross-sectional study. The constant parameters to calculate the DAI were [WC/[22.79+[2.68*BMI]]]*[triglycerides (TG, mmol/L)/1.37]*[1.19/high density lipoprotein-cholesterol (HDL-C, mmol/L)] for males, and [WC/[24.02+[2.37*BMI]]]*[TG(mmol/L)/1.32]*[1.43/HDL-C(mmol/L)] for females. DAI correlated with adipocytes mean area, adipocyte number and adiponectin/leptin ratio. DAI ≥1.065 was independently associated with diabetes, non-alcoholic fatty liver disease, subclinical atherosclerosis, and hypertension. The present study highlights that DAI is associated with early CA independently of adiposity and other risk factors. Since DAI is obtained using accessible parameters, it can be easily incorporated into clinical practice for early identification of AT abnormalities in apparently healthy subjects.

## Introduction

In the last decades, there have been several adverse changes in diet and physical activity [1–3], which led to an increased prevalence of overweight and obesity [4,5]. This situation has become a global emergency due to its association with general mortality [5,6] and cardiometabolic diseases such as non-alcoholic fatty liver disease (NAFLD), hypertension, diabetes, and coronary artery disease (CHD) [2,6,7]. Body mass index (BMI) is the most widely used indicator of excess body weight. However, several authors have questioned the validity of BMI and proposed that adiposity measurements [8–11] or the evaluation of the morpho-functional characteristics of adipose tissue (AT) could be more useful to estimate the risk of developing metabolic abnormalities [12–14].

AT dysfunction is a multi-step process where in early stages, subcutaneous AT fails to store energy properly, which results in enlarged adipocytes (hypertrophic) that promote systemic low-grade inflammation and fat deposition in visceral AT [15]. At later stages, subcutaneous and visceral AT dysfunction leads to impaired systemic metabolism [16,17]. Considering that 10–40% of subjects with normal body weight have been identified with metabolic abnormalities, and 20–30% of those with BMI ≥25 kg/m^2^ appear to be metabolically healthy, several clinical markers have been proposed as subrogated to AT functionality in order to identify cardiometabolic abnormalities [18,19].

Adipocytes synthesize and release adipokines such as adiponectin and leptin, which have metabolic and immunomodulatory activities [20,21]. It has been proposed that a low adiponectin/leptin ratio (ALR) can be considered as a marker of AT dysfunction [22]. On the other hand, Amato et al. [23] developed the visceral adiposity index (VAI), which involves adiposity measurements (BMI and waist circumference [WC]) and biochemical parameters associated with adipose tissue function (triglycerides [TG] and high-density lipoprotein cholesterol [HDL-C]), in order to provide more reliable information about AT functionality. Although VAI has been suggested as a visceral AT function marker [24] this index has been inconsistently associated with cardiometabolic abnormalities [24–26].

Nowadays there are no consistent findings of the usefulness of VAI as a clinical marker of metabolic damage, and no study has investigated the morpho-functionality of adipose tissue of subjects with different VAI. Thus, the aims of the present study were: 1) adapt the VAI for a Hispanic population, 2) establish the adapted index as Dysfunctional Adiposity Index (DAI), based on the analysis of its relationship with subcutaneous AT morpho-functional characteristics, 3) define a cut-off point for DAI based on metabolic abnormalities, and 4) evaluate the usefulness of this DAI cut-off point as a marker of cardiometabolic abnormalities presence, in subjects with no evident cardiovascular disease.

## Methods

Study population

**Figure 1.**
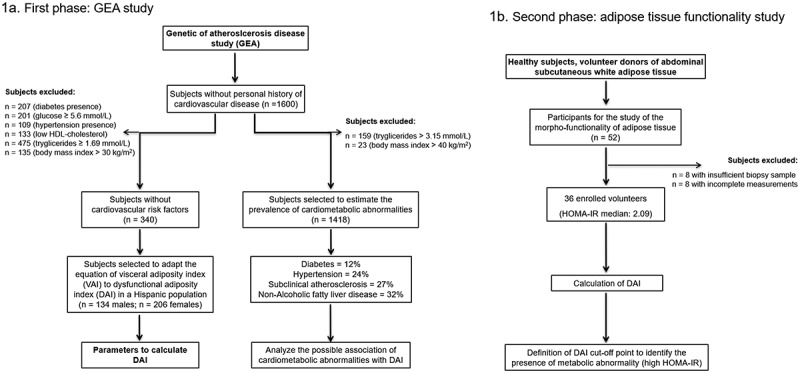
Study flowchart. 1a) GEA study. Diabetes defined as glucose >7.0 mmol/L, hypoglycaemic treatment or previous diagnosis; hypertension as self-reported treatment with antihypertensive medications or systolic/diastolic blood pressure ≥140/90 mmHg; subclinical atherosclerosis as coronary artery calcium >0; non-alcoholic fatty liver disease as spleen-liver attenuation ratio <1.0; and low HDL-cholesterol as values <1.03 mmol/L or <1.29 mmol/L for men or women; respectively. 1b) Study of the adipose tissue functionality. HOMA-IR: homoeostatic model assessment of insulin resistance; HDL: high-density lipoprotein

The present study had two phases. The first phase aimed to adapt VAI to DAI and evaluate its association with cardiometabolic diseases. For that purpose, we selected healthy subjects without cardiovascular risk factors from the Genetics of Atherosclerotic Disease study (Spanish acronym GEA). The GEA study was designed at the National Institute of Cardiology Ignacio Chávez, to examine the genomic basis of CHD and evaluate its relation with traditional and emerging cardiovascular risk factors in an adult Mexican population. The sample included 1200 patients with established premature CHD, and a control group of 1600 subjects aged 35 to 70 years, with no clinical or family history of coronary artery disease, selected from donors attending the blood bank of the National Institute of Cardiology, or recruited by advertisement posters in social service centres from June 2008 through November 2012 [27]. The GEA study participants were extensively characterized, including imaging studies, biochemical measurements, anthropometry, medical history, and sociodemographic and nutritional information collected through validated questionnaires [28]. With the purpose to estimates the constant values needed for the DAI equation, a subgroup of 340 healthy subjects without diabetes and with glucose <5.6 mmol/L, systolic/diastolic blood pressure <140/90 mmHg, TG <1.69 mmol/L, HDL-C ≥ 1.03/1.29 mmol/L for men/women, and BMI <30 kg/m^2^, was selected from the control GEA participants. Additionally, 1418 subjects from the control group of the GEA study were used to estimate the prevalence of cardiometabolic abnormalities and its association with DAI, excluding those participants with TG >3.15 mmol/L or BMI >40 kg/m^2^ that are potential confounders of the original VAI (Figure 1a) [24]. In the second phase of the present study, we analysed subcutaneous AT biopsies and evaluated body composition and circulating adipokines and cytokines in a sample of healthy subjects to validate the usefulness of DAI as a marker of the morpho-functional state of the AT. Briefly, 350 healthy individuals without cardiovascular disease, dyslipidemia (TG >3.15 mmol/L), infectious disease, diabetes, cancer, or any autoimmune disease, were invited through phone calls and personal interviews (from February 2018 to May 2019), to participate in the study to investigate the morpho-functionality of their AT. Fifty-two healthy subjects voluntarily agreed to participate and were enrolled, but only 36 participants were included in the analyses due to inadequate subcutaneous AT biopsy, biochemical measurements, anthropometry, or incomplete clinical information (Figure 1b). Both studies were approved by the research and ethics committee and performed following the guidelines of the Helsinki Declaration. All participants voluntarily signed the institutional informed consent form.

Clinical and anthropometrical evaluation

Participants in both studies were interviewed by trained research staff and completed questionnaires detailing medical history, demographic characteristics, CHD history, medication, alcohol, and tobacco use. Height, weight, and WC were measured; BMI was calculated based on weight (kg) divided by height (m^2^). WC was measured at the midpoint between the top of the iliac crest and the lower margin of the last palpable rib in the midaxillary line, with the patient in a standing position. After a 10 minutes rest period, blood pressure was measured three times; the average of the second and third measurements was used for the analysis.

Biochemical measurements

In both studies, blood samples were obtained from an antecubital vein of each patient after a 12-h overnight fast and 20 min in a sitting position. Plasma glucose, total cholesterol, TG, and HDL-C were measured using standardized procedures (Roche Diagnostics, Mannheim, Germany). Low-density lipoprotein cholesterol was estimated using the De Long et al. formula [29]. Accuracy and precision of lipid measurements in our laboratory are under periodic surveillance by the Center for Disease Control and Prevention service (Atlanta, GA, USA). Inter-assay coefficients of variation were <6% for all these assays. High-sensitivity C-reactive protein (hs-CRP) was determined by immunonephelometry on a BN ProSpec nephelometer (Dade Behring, Germany), according to the manufacturer’s method, with intra and inter-assay variation coefficients below 3%. Adiponectin, leptin, interleukin 6 (IL-6), interleukin 1β (IL-1β), monocyte chemoattractant protein-1 (MCP-1), plasminogen activator inhibitor-1 (PAI-1), and insulin were quantified using a Bio-Plex system (Bio-Rad Inc, USA), with intra- and inter-assay variation coefficients below 4% and 5% (respectively), for all these assays. ALR was calculated as an indicator of AT functionality [22,30]. The homoeostatic model assessment of insulin resistance (HOMA-IR) index was calculated using the formula: HOMA-IR = (Glucose [mmol/l] × Insulin [µIU/l])/(22.5).

Computed tomography (CT)

Participants from the GEA study control group underwent CT to determine the presence of fatty liver or coronary artery calcium score >100 Agatston units as a subrogated of subclinical atherosclerosis. CT is a validated method for measuring coronary artery calcium [31] and NAFLD [32]. CT of the chest was performed using a 64-channel multi-detector helical computed tomography system (Somatom Sensation, Siemens), and experienced radiologists interpreted images. Scans were examined to determine coronary artery calcification scores using the Agatston method [31]. A single CT scan slice obtained at T11–T12 or T12–L1 level was used to determine the liver and spleen attenuation [32]

Electrical bioimpedance

Body composition was determined in participants of the second phase of the study, using a 6-frequency electrical bioimpedance analyser (InBodyS10, Korea), in the supine position. Total body fat percentage and visceral fat area (cm^2^) were obtained. All study participants were asked to have at least 4 hours of fasting before measurement and have no metal or electronic equipment with them during the test.

Cardiometabolic abnormalities definition

In all subjects, diabetes was defined as glucose >7.0 mmol/L, hypoglycaemic treatment or previous diagnosis [33], NAFLD as spleen-liver attenuation ratio <1.0 [32], hypertension as self-reported treatment with antihypertensive medications or systolic/diastolic blood pressure ≥140/90 mmHg [34] and subclinical atherosclerosis as coronary artery calcium >100 Agatston units [31].

Adipose tissue biopsies

In the 52 healthy volunteers enrolled in the study of AT morpho-functionality, a subcutaneous white AT sample was obtained from periumbilical fat, with surgical technique under local anaesthesia (2% lidocaine), and after an overnight fast [35]. Biopsies were immediately rinsed with sterile saline, and visible blood vessels were removed. AT biopsies were fractionated into three pieces. Two pieces were immediately frozen in liquid nitrogen. The last piece was immediately fixed in PBS-buffered 4% paraformaldehyde for histological analyses.

Analysis of the number and mean area of adipocytes

After 24 hours, fixed tissues were dehydrated in ethanol, cleared in xylene, embedded in paraffin, sectioned at 4 µm, and stained with haematoxylin and eosin. Digital images were obtained using a digital camera (Leica ICC50 HD) coupled to Leica DM750 microscope using a 20X lens at a resolution of 2048 × 1536 pixels using LAS EX V 3.0 software (Leica Microsystems, Switzerland). Five fields of the captured images were taken per slide in varying parts of the fat biopsies of each patient. Adipocytes size was measured by converting pixels into microns using Adiposoft (ImageJ) software with the following parameters: minimum diameter 10 µm, maximum diameter 1000 µm, and microns per pixel 0.439 [36] The mean adipocytes area (µm^2^) and the number of adipocytes (per field) were determined in a blinded manner taking into account all the adipocytes of each patient’s five fields. After completing the automated analyses of the number and area of adipocytes measurements, each value was checked manually to ensure that it represented a single adipocyte to prevent errors that may have occurred during automated analyses.

Dysfunctional adiposity index

The original VAI formula is calculated separately for men or women and includes two sections [23].; the first section represents the proportion of central AT through WC with respect to BMI as a measure of body fat. With linear regression analysis between both adiposity markers, the constants of the intercepts and the slopes can be determined, which are included in the formula: WC/[constant intercept + (slope constant * BMI)]. The second section represents AT function through TG and HDL-C plasma concentration. In this case, median values of TG (mmol/L) and HDL-C (mmol/L) from healthy subjects without cardiometabolic risk factors were used as reference. Thus, the final equation for DAI estimation by gender is represented as follows:

$$DAI=\left(\frac{WC}{constantintercept+\left(constantslope*BMI\right)}\right)\;\left(\frac{TG}{\begin{array}{c} TGmedian\\ reference \end{array}}\right)\left(\frac{\begin{array}{c} HDL-Cmedian\\ reference \end{array}}{HDL-C}\right)$$

Statistics

Data are presented as means ± standard deviation, median (interquartile range), and the number of subjects (percentages). HOMA-IR was calculated in participants of the morpho-functionality AT study, and subjects stratified according to the median value (above or below). A comparison of these groups was made with Student’s t, Mann–Whitney U, or Chi-square tests, respectively. Spearman coefficient correlation was calculated to evaluate the relationship of DAI with inflammatory and morpho-functional characteristics of AT. The discriminative power of the DAI and other adiposity measurements to identify subjects with HOMA-IR above the median was examined by calculation of the area under the receiver operating characteristic (ROC) curve (area under the curve [AUC]); the optimal cut-off value was determined by the maximal Youden index. The association of high DAI values with cardiometabolic abnormalities was considered in the control group of the GEA study, through a multiple stepwise logistic regression analysis adjusted to potential confounders; values are shown as odds ratio (95% interval of confidence). All p values <0.05 were considered statistically significant. The statistical analyses were performed using 15.0 software SPSS (Chicago, IL, USA) and SAS JMP ® Trial version 15.1.0 (Cary, NC, USA).

## Results

The control group of the GEA study included 1600 participants without CHD (50.8% women), with a mean age of 53 ± 9 years, and a BMI of 28.4 ± 4.3 Kg/m^2^. To estimate the DAI, the intercept and slope constants of the VAI formula were recalculated in a subgroup of the GEA study of 340 subjects without cardiovascular risk factors (134 males and 206 females). The mean age of this subgroup was 52 ± 10 years, BMI = 25.1 ± 2.5 Kg/m^2^, WC = 85.8 ± 8.5 cm, TG = 1.15 (0.92–1.4) mmol/L, and HDL-C = 1.47 (1.34–1.70) mmol/L. The linear relationship between WC and BMI shows a significant positive correlation between these parameters in both, males (R^2^ = 0.667, p < 0.001; linear equation: WC = 22.79 + 2.68*BMI) and females (R^2^ = 0.584, p < 0.001; linear equation: WC = 24.02 + 2.37*BMI). Median values of TG and HDL-C were also calculated in men (1.37 mmol/L and 1.19 mmol/L) and women (1.32 mmol/L and 1.43 mmol/L). These parameters were used to estimate DAI as following:

$$DAI\;male\;=\;\left(\frac{WC}{22.79+(2.68*BMI)}\right)\;\left(\frac{TG}{1.37}\right)\;\left(\frac{1.19}{HDL-C}\right)$$

$$DAI\;female\;=\;\left(\frac{WC}{24.02+(2.37*BMI)}\right)\;\left(\frac{TG}{1.32}\right)\;\left(\frac{1.43}{HDL-C}\right)$$

The study of the AT functionality includes 36 healthy individuals (72% women), average age of 57 ± 9 years, and a BMI of 26 ± 4 kg/m^2^. Supplementary Table A1 shows the clinical and biochemical characteristics of these subjects stratified according to the HOMA-IR median value (2.09). When components of the DAI were compared, we did not find statistical differences in BMI, WC, and HDL-C; however, TG was significantly higher among the subjects with higher HOMA-IR values. No differences were found between groups regarding age, percentage of total fat mass, visceral fat area, glucose, total cholesterol, low-density lipoprotein cholesterol, diastolic and systolic blood pressure, or tobacco use. The systemic inflammation markers and morpho-functional characteristics of the AT are shown in Supplementary Table A2. Compared with subjects with HOMA-IR <2.09, those with higher HOMA-IR values were characterized to have a trend towards higher hs-CRP (+128.3%), IL-1β (+55.1%), IL-6 (+27.6%), MCP-1 (+8.0%), and PAI-1 (+7.2%) values; however, a statistical difference was found only in hs-CRP serum levels. High HOMA-IR subjects also showed higher circulating leptin levels (+134.6%), and although they only showed a trend towards lower levels of adiponectin (−12.1%), it was observed that the ALR was significantly lower (−56.1%) than subjects with HOMA-IR below the median. The morphological evaluation of the AT showed that subjects with high HOMA-IR values displayed 44.7% bigger adipocytes mean area and 25.9% fewer adipocyte-count per field, which denotes hypertrophy that was confirmed by an 84.3% higher ratio of area/number of adipocytes. Additionally, the DAI also showed significantly higher values (+107.9%) among subjects with HOMA-IR above the median.

**Figure 2.**
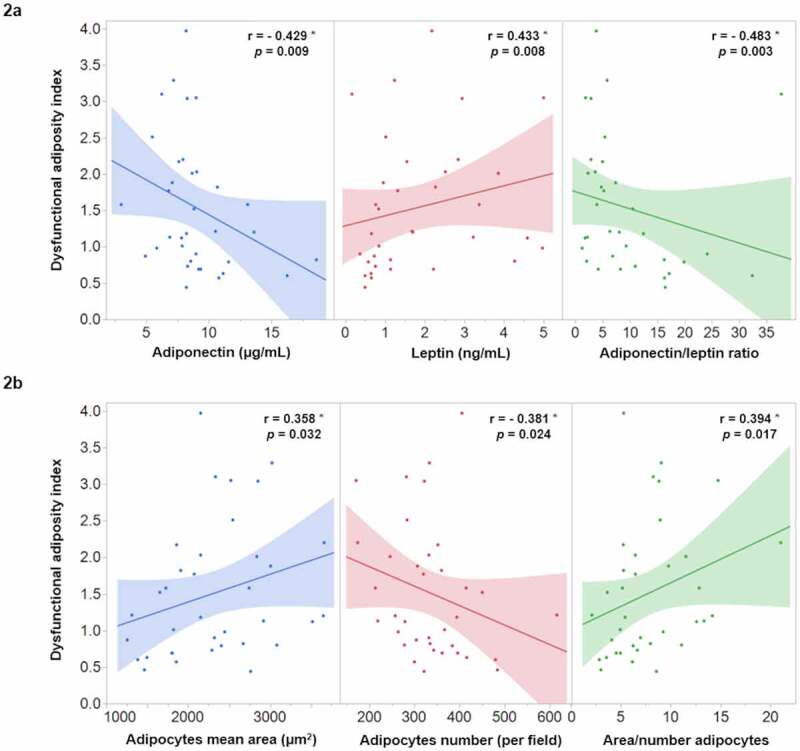
Correlation of dysfunctional adiposity index with functional (2a) and morphological (2b) characteristics of adipose tissue in 36 healthy subjects. * Spearman correlation coefficient, the shaded area represents the 95% confidence interval

**Figure 3.**
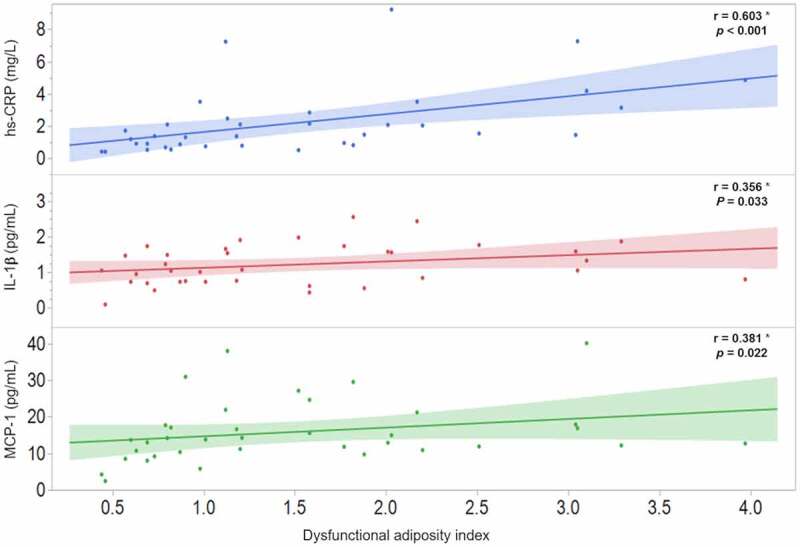
Correlation of dysfunctional adiposity index with systematic inflammation markers in 36 healthy subjects. hs-CRP: high-sensitivity C-reactive protein; IL: interleukin; MCP-1: monocyte chemoatractant protein-1. * Spearman correlation coefficient, shaded area represents the 95% confidence interval

To investigate the possible association of DAI with the morpho-functional characteristics of AT, a Spearman coefficient correlation was carried out in the 36 participants of the AT functionality study. The results revealed that DAI is strongly and significantly associated with both, functional (Figure 2a) and morphological (Figure 2b) AT characteristics. Moreover, the possible relationship of DAI with systemic inflammatory markers was analysed and the results shown the strong association of DAI with hs-CRP (r = 0.603; *p* < 0.001), IL-1β (r = 0.356; *p* = 0.033), and MCP-1 (r = 0.381; *p* = 0.022) (Figure 3).

**Figure 4.**
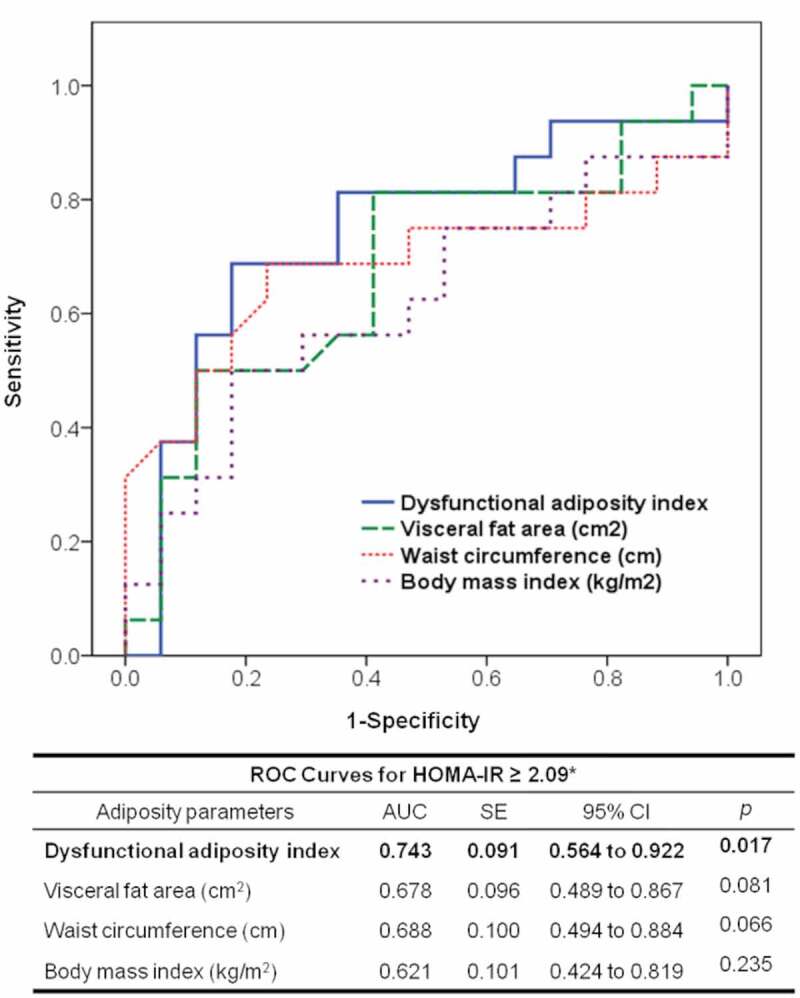
Receiver operating characteristic (ROC) curve to predict the homoeostatic model assessment of insulin resistance (HOMA-IR) ≥2.09. AUC: area under the curve; SE: standard error; CI: confidence interval. * This represents the median value in 36 healthy subjects

Participants of the AT functionality study were analysed in a ROC curve model to establish a DAI cut-off value to identify subjects with metabolic abnormalities, using HOMA-IR above the median as an indicator of metabolic abnormality. Figure 4 shows that compared to BMI (AUC: 0.621; *p* = 0.235), WC (AUC: 0.688; *p* = 0.066), and visceral fat (AUC: 0.678; *p* = 0.081) the area under the curve of DAI was the only significantly associated with elevated HOMA-IR (AUC: 0.743; *p* = 0.017). Additionally, according to the Youden index, we found that a DAI value >1.065 could be useful to identify subjects with metabolic abnormalities with a sensitivity of 81% and specificity of 65%.

**Figure 5.**
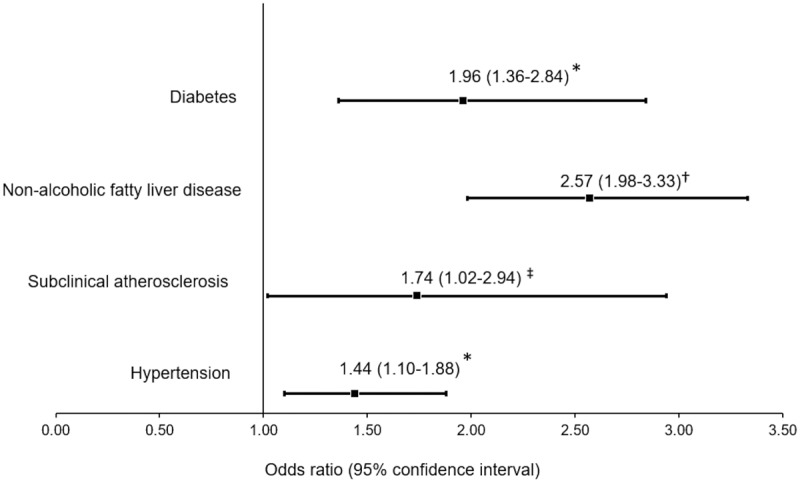
Association of high dysfunctional adiposity index (>1.065) with cardiometabolic abnormalities in 1418 subjects without a history of cardiovascular disease. Diabetes is defined as glucose >7.0 mmol/L, hypoglycaemic treatment or previous diagnosis; non-alcoholic fatty liver disease as spleen-liver attenuation ratio <1.0; subclinical atherosclerosis as coronary artery calcium >0, and hypertension as self-reported treatment with antihypertensive medications or systolic/diastolic blood pressure ≥140/90 mmHg. * Multiple logistic regression analysis adjusted by age, sex, and tobacco use

To evaluate the usefulness of the DAI cut-off point in identifying cardiometabolic abnormalities, we determined the prevalence of diabetes (12%), NAFLD (32%), subclinical atherosclerosis (27%), and hypertension (24%) in subjects without history of CHD of the GEA study (Table 1). As shown in Figure 5, logistic regression analysis indicates that DAI values greater than 1.065 were significantly associated with higher probability of diabetes (+96%), NAFLD (+157%), subclinical atherosclerosis (+74%), and hypertension (+44%); even after adjusting for well-established cardiovascular risk factors.

**Table 1.** Clinical and biochemical characteristics of subjects (n = 1418) without history of coronary arterial disease

Females, n (%)	744 (52%)
Age (years)	54 ± 9
Body mass index (kg/m^2^)	28 ± 4
Waist circumference (cm)	94 ± 11
Diastolic blood pressure (mmHg)	72 ± 9
Systolic blood pressure (mmHg)	117 ± 18
Tobacco use, n (%)	312 (22)
Total cholesterol (mmol/L)	4.9 ± 0.9
LDL-C (mmol/L)	3.0 ± 0.7
HDL-C (mmol/L)	1.2 ± 0.3
Triglycerides (mmol/L)	1.5 (1.2–2.10)
Glucose (mmol/L)	5.3 ± 1.6
Insulin (µUI/L)	16 (12–22)
HOMA-IR	3.8 (2.6–5.5)
Dysfunctional adiposity index ≥1.065, n (%)	896 (63)

Values expressed as number (percentage), mean ± standard deviation or median (interquartile range). LDL-C: low-density lipoprotein cholesterol; HDL-C: high-density lipoprotein cholesterol; HOMA-IR: homoeostatic model assessment of insulin resistance.

## Discussion

The efficacy of anthropometry and various imaging techniques, as markers of cardiometabolic health, has been extensively debated since those measurements are unable to evaluate AT function [10,11]. The VAI has been considered an indicator of AT functionality, but its association with metabolic derangements is inconsistent [24]. These inconsistencies can occur from applying VAI to subjects of different ethnicity and clinical characteristics of the studied populations for which it was described. The present study remarks on the importance of adapting the VAI equation to the clinical features of a particular population with respect to its ethnicity, or otherwise and with the use of a multicenter study, establishes a coefficient factor in the equation to adjust for ethnicity. Furthermore, our data lead us to establish this equation as a more general index by analysing, for the first time, its association with morpho-functional AT and systemic inflammation markers. The clinical usefulness of the DAI was proved at general population level and showed a strong and independent association with cardiometabolic abnormalities. Our results suggest that the DAI can be considered a practical and economical tool to identify increased cardiovascular risk in otherwise apparently healthy subjects. Although the foregoing statement must be verified in prospective studies that include larger populations, its confirmation could indicate that the use of DAI in clinical practice could identify timely and prevent cardiometabolic complications related to the AT dysfunction.

A group of individuals with excess body weight, identified through anthropometry and imaging techniques, seemed to have relative protection against negative metabolic outcomes, suggesting that AT functionality rather than fat mass may be the key factor in the pathophysiology of metabolic and cardiovascular diseases [8–14]. Considering that the VAI was based on Caucasian population and that parameters included in the equation have multiple discrepancies among various ethnicities, it is important to adapt the VAI according to each ethnicity. The present analysis support that by showing that in contrast with the original VAI equation, the DAI equation shows a different pattern in the intercept and slope constants, when WC and BMI were analysed as the expression of AT distribution. Similarly, TG and HDL-C median values are different in Hispanic population, as previously reported [37]. Moreover, in an additional analysis using the original parameters reported by Amato et al. [23], to estimate VAI in our population, we found lower and non-significant correlation of VAI with leptin (r = 0.288; *p* = 0.093), adipocytes size (r = 0.262; *p* = 0.128), adipocytes number (r = −0.228; *p* = 0.187), IL-1β (r = 0.205; *p* = 0.238), and MCP-1 (r = 0.323; *p* = 0.059) as compared with the observed with the DAI (Figure 2 and Figure 3). In line with this, two previous studies in Asian populations adapted the VAI to their clinical characteristics obtaining a significant association with a higher risk of cardiometabolic diseases [25,26].

The ALR has been proposed as an emerging biomarker of AT dysfunction that correlates with insulin resistance, metabolic abnormalities, and inflammatory cytokines [22,30]. On the other hand, reduced adipocytes cell number and enlarged size have been considered morphological indicators of hypertrophic adipocytes that play a role in obesity-related cardiovascular and metabolic abnormalities [15–17]. Data of the present study indicate that ALR significantly correlates with subcutaneous adipocytes number (r = 0.525; *p* = 0.001) and area (r = −0.431; *p* = 0.009), as well as with the relation of area/number of adipocytes (r = −0.529; *p* = 0.001) as indicator of morphologically abnormal adipocytes. Moreover, the analysis of the present study highlights that DAI was strongly correlated with these morpho-functional SAT characteristics (Figure 2) and higher systemic low-grade inflammation (Figure 3). It is important to underline that our study analysed SAT from healthy subjects, representing the normal physiological buffer for excess energy intake. When the storage capacity of SAT is exceeded, or its ability to generate new adipocytes is impaired, TG begins to accumulate in tissues outside the SAT, such as VAT, skeletal muscle, and liver [15,38]. Thus, it can be hypothesized that in the progression of cardiometabolic alterations, the earlier stages of AT dysfunction comprise the impairment in SAT expansion and activation of inflammatory macrophages, reflected by a low number of adipocytes, hypertrophy, and the presence of low-grade systemic inflammation (MCP-1, IL-1β, and hs-CRP). This hypothesis is supported by previous studies showing a differential response of VAT and SAT to obesity-induced inflammation [14,39,40]. Together, these data also support the notion of renaming the morpho-functional AT index as DAI since VAI rater refers only to VAT, which becomes dysfunctional at later stages in the process of AT derangements.

The present study shows that compared with the DAI, measurements of BMI, WC, and visceral fat areas were not significantly associated with higher HOMA-IR values. Additionally, the results showed that DAI values higher than 1.065 were strongly and independently associated with the presence of cardiometabolic diseases (Figure 5). These data suggest that in subjects with BMI ≤ 40 kg/m^2^ and TG ≤ 3.15 mmol/L, the DAI may be a useful tool to identify earlier changes in the morpho-functionality of AT before clinical manifestations.

Our study has several strengths. 1) The healthy subjects from the GEA study to adapt the VAI to DAI were thoroughly characterized, allowing us to calculate constants reliably for the equation. 2) Unlike other studies that include subjects with morbid obesity and other comorbidities, participants of the present study had BMI similar to the general population (～27 kg/m^2^) and were apparently healthy, which could reflect the early stages of AT dysfunction. 3) We evaluated the usefulness of a DAI cut-off in a group of subjects of the GEA study that included participants without clinical cardiovascular disease history. On the other hand, subjects that underwent biopsy in the present study were healthy, so obtaining VAT could represent an unnecessary risk. Thus, a potential limitation is the lack of VAT samples, which would allow us to discriminate the participation of each fat component during the early stages of AT dysfunction. Another limitation is that we could not measure specific markers, gene, or protein expression from the AT sample. However, several studies have demonstrated that circulating adipose tissue-specific cytokines (such as adiponectin and leptin), can reflect the functional status of the adipocytes [20,21]. Finally, due to the cross-sectional nature of the present study, we could not determine the causality of the associations.

Although excess body weight and the associated diseases have been globally and simultaneously increasing in their prevalence, AT dysfunction appears to be the key factor in the pathophysiology of obesity-related chronic metabolic and cardiovascular diseases. Nowadays, there are no useful and straightforward clinical markers that can identify early abnormalities in AT from subjects with increased risk for developing cardiometabolic disorders. The results of this study allowed us to establish the constant parameters needed to calculate DAI as an index of dysfunctional adipocytes in a Hispanic population. This index is based on accessible routine parameters evaluated in clinical practice and could be useful to identify early abnormalities of AT. The latter was confirmed by proving the association of the DAI with the morphological and functional characteristics of the SAT and with the low-grade systemic inflammation of apparently healthy subjects. Furthermore, our results highlight that regardless of adiposity and other risk factors, DAI values greater than 1.065 could be associated with the presence of early cardiometabolic abnormalities.

## Supplementary Material

Supplemental Material

## Supporting information

Table A1. Clinical and biochemical characteristics of apparently healthy subjects stratified according to the HOMA-IR median value.

Table A2. Systemic inflammation markers and morpho-functional characteristics of adipose tissue in apparently healthy subjects.
