# Supplementary material for: Dysfunctional adiposity index as a marker of adipose tissue morpho-functional abnormalities and metabolic disorders in apparently healthy subjects
Source: Adipocyte. 2021 Mar 16;10(1):142–52. doi: 10.1080/21623945.2021.1893452 (PMC7971216; doi:10.1080/21623945.2021.1893452)
Supplement: Supplemental Material [file KADI_A_1893452_SM3163.docx]

**Supplementary Table A1.** Clinical and biochemical characteristics of 36 apparently healthy subjects stratified according to the HOMA-IR median value (2.09).

|  | HOMA-IR < 2.09  n=18 | HOMA-IR ≥ 2.09  n=18 | *p* * |
| --- | --- | --- | --- |
| Females, n (%) | 12 (66) | 14 (77) | 0.457 |
| Age (years) | 57 ± 10 | 56 ± 7 | 0.870 |
| Body mass index (Kg/m^2^ ) | 26 ± 3 | 27 ± 5 | 0.258 |
| Waist circumference (cm) | 85 ± 7 | 90 ± 10 | 0.085 |
| % Total fat mass | 34 ± 8 | 39 ± 8 | 0.063 |
| Visceral fat area (cm^2^) | 96 (82-134) | 144 (115-180) | 0.081 |
| Triglycerides (mmol/L) | **1.3 (0.9-1.4)** | **2.1 (1.4-2.5)** | **0.007** |
| HDL-C (mmol/L) | 1.3 ± 0.3 | 1.1 ± 0.3 | 0.259 |
| Glucose (mmol/L) | 5.3 ± 0.4 | 5.6 ± 0.5 | 0.053 |
| Total cholesterol (mmol/L) | 4.4 ± 0.9 | 4.9 ± 1.3 | 0.133 |
| LDL-C (mmol/L) | 2.6 ± 0.72 | 3.1 ± 2.2 | 0.171 |
| Insulin (µUI/L) | **6.1 (4.6-8.2)** | **11.1 (8.3-12.7)** | **<0.001** |
| HOMA-IR | **1.5 (0.9-1.9)** | **2.7 (2.1-3.1)** | **<0.001** |
| Diastolic blood pressure (mmHg) | 70 ± 8 | 73 ± 8 | 0.247 |
| Systolic blood pressure (mmHg) | 112 ± 16 | 120 ± 15 | 0.128 |
| Tobacco use, n (%) | 4 (22) | 3 (16) | 0.674 |

Values expressed as number (percentage), mean ± standard deviation or median (interquartile range).* Student t, U Mann-Whitney or Chi-square test. HOMA-IR: homeostatic model assessment of insulin resistance; HDL-C: high density lipoprotein cholesterol; LDL-C: low density lipoprotein cholesterol.

**Supplementary Table A2.** Systemic inflammation markers and morpho-functional characteristics of adipose tissue in 36 apparently healthy subjects stratified according to the HOMA-IR median value (2.09).

|  |  | HOMA-IR < 2.09  n=18 | HOMA-IR ≥ 2.09  n=18 | *p ** |
| --- | --- | --- | --- | --- |
| Systemic inflammation markers | **hs-CRP (mg/L)** | **0.92 (0.7-1.4)** | **2.1 (1.4-3.2)** | **0.005** |
|  | IL-1β (pg/mL) | 0.98 ( 0.7-1.5) | 1.52 ( 0.8-1.7) | 0.277 |
|  | IL-6 (pg/mL) | 2.9 (2.2-4.1) | 3.7 (2.5-4.2) | 0.467 |
|  | MCP-1 (pg/mL) | 13.8 (10.3-16.6) | 14.9 (11.2-21.9) | 0.255 |
|  | PAI-1 (ng/mL) | 6.9 (5.4-7.5) | 7.4 (5.6-9.2) | 0.174 |
| Morpho-functional adipose tissue characteristics | Adiponectin (µg/mL) | 9.1 (8.1-11.1) | 8.0 (7.0-8.6) | 0.062 |
|  | **Leptin (ng/mL)** | **0.81 (0.6-1.7)** | **1.9 (0.9-3.3)** | **0.029** |
|  | **Adiponectin/leptin ratio** | **9.8 (5.6-17.1)** | **4.3 (2.2-7.3)** | **0.015** |
|  | **Adipocytes mean area (µm^2^)** | **1899 ± 420** | **2734 ± 571** | **< 0.001** |
|  | **Adipocytes number (per field)** | **386 ± 83** | **286 ± 65** | **< 0.001** |
|  | **Area/number of adipocytes** | **5.1 (3.6-6.4)** | **9.4 (8.2-12.7)** | **< 0.001** |
|  | **Dysfunctional adiposity index** | **0.89 (0.6-1.2)** | **1.85 (1.1-2.5)** | **0.007** |

Values expressed as mean ± standard deviation or median (interquartile range). *Student t or U Mann-Whitney test. HOMA-IR: homeostatic model assessment of insulin resistance; hs-CRP: high-sensitivity C-reactive protein, IL: interleukin, MCP-1: monocyte chemoattractant protein-1, PAI-1: plasminogen activator inhibitor-1.
